# Bis(4-hy­droxy-*N*-isopropyl-*N*-methyl­trypt­ammo­nium) fumarate: a new crystalline form of miprocin

**DOI:** 10.1107/S2056989020002923

**Published:** 2020-03-10

**Authors:** Andrew R. Chadeayne, Duyen N. K. Pham, James A. Golen, David R. Manke

**Affiliations:** a CaaMTech, LLC, 58 East Sunset Way, Suite 209, Issaquah, WA 98027, USA; b University of Massachusetts Dartmouth, 285 Old Westport Road, North Dartmouth, MA 02747, USA

**Keywords:** crystal structure, tryptamines, indoles, hydrogen bonding

## Abstract

The title compound, bis­(4-hy­droxy-*N*-isopropyl-*N*-methyl­tryptammonium) (4-HO-MiPT) fumarate (systematic name: bis­{[2-(4-hy­droxy-1*H*-indol-3-yl)eth­yl](meth­yl)propan-2-yl­aza­nium} but-2-enedioate), 2C_14_H_21_N_2_O^+^·C_4_H_2_O_4_^2−^, has a singly protonated tryptammonium cation and one half of a fumarate dianion in the asymmetric unit. The tryptammonium and fumarate ions are held together in one-dimensional chains by N—H⋯O and O—H⋯O hydrogen bonds. These chains are a combination of *R*_4_^2^(20) rings, and *C*_2_^2^(15) and *C*_4_^4^(30) parallel chains along (110). They are further consolidated by N—H⋯π inter­actions. There are two two-component types of disorder impacting the tryptammonium fragment with a 0.753 (7):0.247 (7) occupancy ratio and one of the fumarate oxygen atoms with a 0.73 (8):0.27 (8) ratio.

## Abstract 2

A new form of the synthetic psychedelic miprocin, a psilocin derivative, is reported. The title compound has a single protonated tryptammonium cation and one half of a fumarate in the asymmetric unit. The ions are held together in an elaborate set of rings and chains along (110).

## Chemical context   

A wide variety of naturally occurring organisms, including over 200 species of ‘magic’ mushrooms, contain psychoactive tryptamine compounds (Stamets, 1996 ▸). Of these compounds, psilocybin has received the most scientific and commercial attention because of recent studies demonstrating its potential for treating mood disorders including addiction, anxiety, depression and PTSD (Johnson & Griffiths, 2017 ▸; Carhart-Harris & Goodwin, 2017 ▸).

Although psilocybin is currently classified as a schedule I drug, the US Food and Drug Administration recently designated treatment using psilocybin a ‘breakthrough therapy’. This status has allowed psilocybin to be administered in clinical trials to treat major depressive disorder and treatment-resistant depression (Feltman, 2019 ▸). Recent reports also suggest that psychedelic microdosing can improve memory, attention and sociability (Cameron, *et al.* 2020 ▸).

Psilocybin is one of at least ten psychoactive tryptamines present in ‘magic’ mushrooms, with natural psilocybin analogs being identified as recently as 2019 (Lenz *et al.*, 2017 ▸; Blei *et al.*, 2020 ▸). Variations in the three-dimensional structure of these natural analogs (as well as synthetic analogs) correlate with differences in their cellular and clinical pharmacology through their structure–activity relationship (SAR) (Nichols, 2018). Understanding the SAR for psilocybin analogs requires the attainment of accurate information about each compound’s 3D structure, best provided through single crystal X-ray diffraction.

Last year, we reported the structure of 4-acet­oxy-*N*,*N*-di­methyl ­tryptamine (4-AcO-DMT) fumarate, which is a syn­thetic analogue of psilocybin. The compound crystallized as a one-to-one tryptammonium/hydro­fumarate salt (Chadeayne *et al.*, 2019*c* ▸). We later synthesized bis­(4-acet­oxy-*N*,*N*-di­methyl­tryprammonium)­fumarate by treating 4-AcO-DMT fumarate with one half equivalent of lead(II) acetate, precipitating half of the fumarate dianions as lead(II) fumarate (Chadeayne, Golen & Manke, 2019*a* ▸).
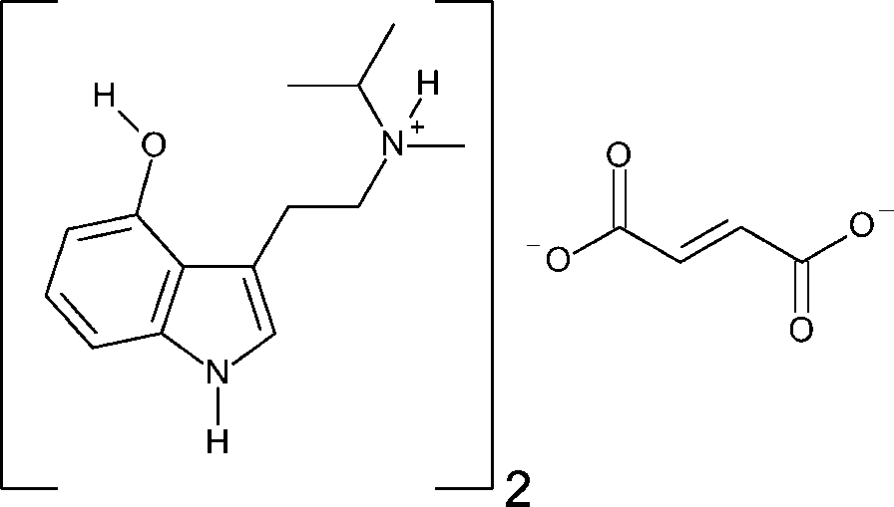


4-Hy­droxy-*N*-methyl-*N*-iso­propyl­tryptamine (4-HO-MiPT), aka ‘miprocin’, is a psilocybin analogue. Its synthesis was first reported in 1981 by Repke and co-workers (Repke *et al.*, 1981 ▸); its psychedelic effects were later described in collaboration with Alexander Shulgin (Repke *et al.*, 1985 ▸). Miprocin is reported to produce an experience that is both relaxing, stoning and mildly sedating with a marked physical stimulation that distinguishes it from related substances such as psilocybin mushrooms. In a report last year, we presented the first structure of 4-HO-MiPT (Chadeayne, Pham *et al.*, 2019*a* ▸), which crystallizes as the hydro­fumarate monohydrate. Herein we report the reaction of this salt with lead(II) acetate to generate the 4-hy­droxy-*N*-isopropyl-*N*-methyl­tryptam­in­ium/fumarate compound in a 2:1 ratio. The solid state structure of the new salt is presented here.

## Structural commentary   

**Figure 1:**
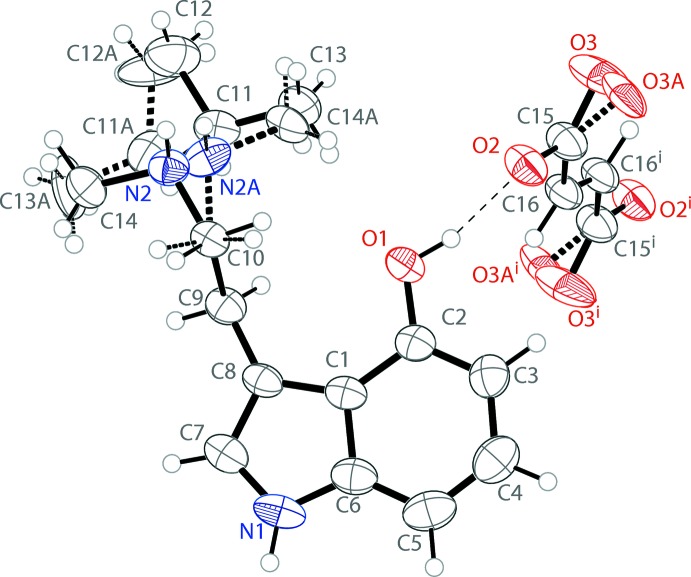
The mol­ecular structure of bis­(4-hy­droxy-*N*-isopropyl-*N*-methyl­tryptammonium)­fumarate, showing the atom labeling. Displacement ellipsoids are drawn at the 50% probability level. Hydrogen bonds are shown as dashed lines. Symmetry code: (i) 1 − *x*, 1 − *y*, 1 − *z*.

The asymmetri unit of bis­(4-hy­droxy-*N*-isopropyl-*N*-methyl­trypt­ammo­nium) fumarate contains one tryptammonium cation and one half of a fumarate dianion (Fig. 1 ▸). The cation possesses a near planar indole, with mean deviation from planarity of 0.014 Å. The methyl­amino group is turned away from this plane, with a C1—C8—C9—C10 torsion angle of −74.2 (2)°. The *N*-isopropyl-*N*-methyl­trypt­ammo­nium group is disordered over two orientations in a 0.753 (7):0.247 (7) ratio, with the two moieties related to each other by a pseudo-mirror operation. In solution, the two conformations are most likely inter­converting into each other by rapid de- and reprotonation. One oxygen atom of the half fumarate anion is also disordered over two positions in a 0.73 (8):0.27 (8) ratio. Half of the fumarate dianion is present in the asymmetric unit, with the other half generated by inversion; it is slightly distorted from planarity with r.m.s. deviations of 0.020 and 0.070 Å for the two components. The carboxyl­ate unit is fairly delocalized, with C—O distances ranging from 1.251 (10) to 1.284 (2) Å.

## Supra­molecular features   

**Figure 2:**
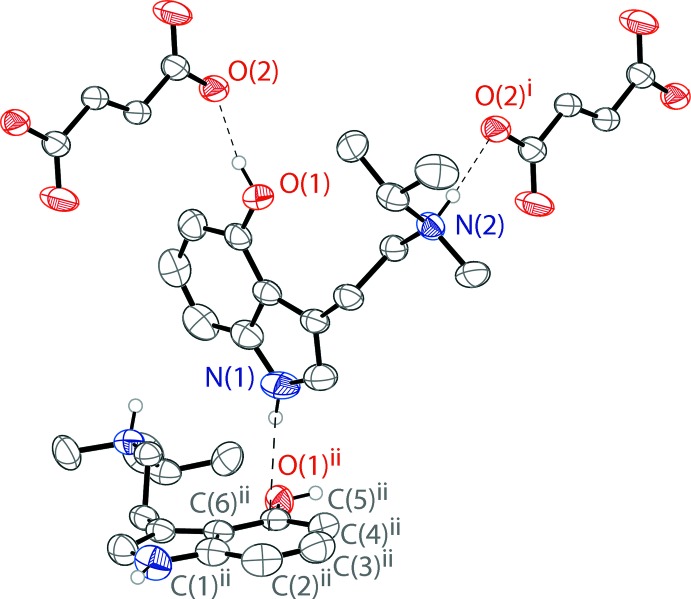
The hydrogen bonding of the tryptammonium cation in the structure of the title compound (Table 1 ▸), with hydrogen bonds shown as dashed lines. There is also an N—H⋯π inter­action shown. Displacement ellipsoids are drawn at the 50% probability level. Hydrogen atoms not involved in hydrogen bonds are omitted for clarity. Symmetry codes: (i) 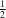 − *x*, 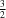 − *y*, 1 − *z*, (ii) 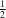 − *x*, −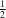 + *y*, 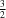 − *z*.

**Figure 3:**
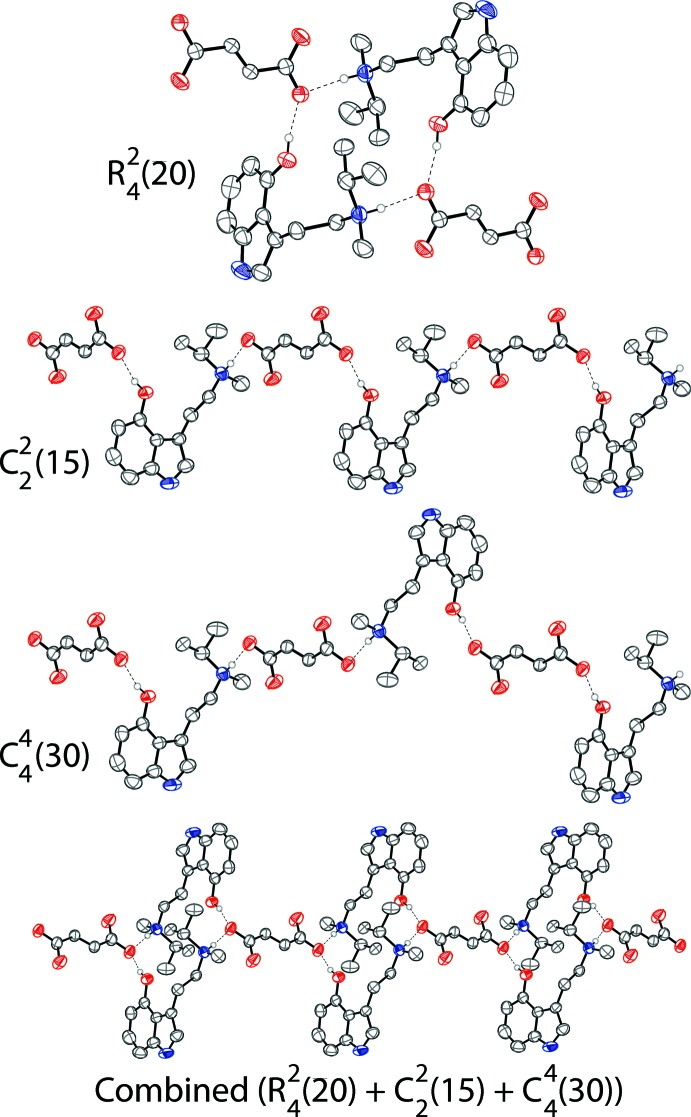
The hydrogen-bonding network along (110), which consists of 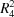
(20) rings that are joined together by two parallel 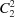
(15) and 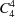
(30) chains. The three components described in graph-set notation and the combined chain are shown. Displacement ellipsoids are drawn at the 50% probability level. Hydrogen atoms not involved in hydrogen bonding are omitted for clarity. Hydrogen bonds are shown as dashed lines.

**Figure 4:**
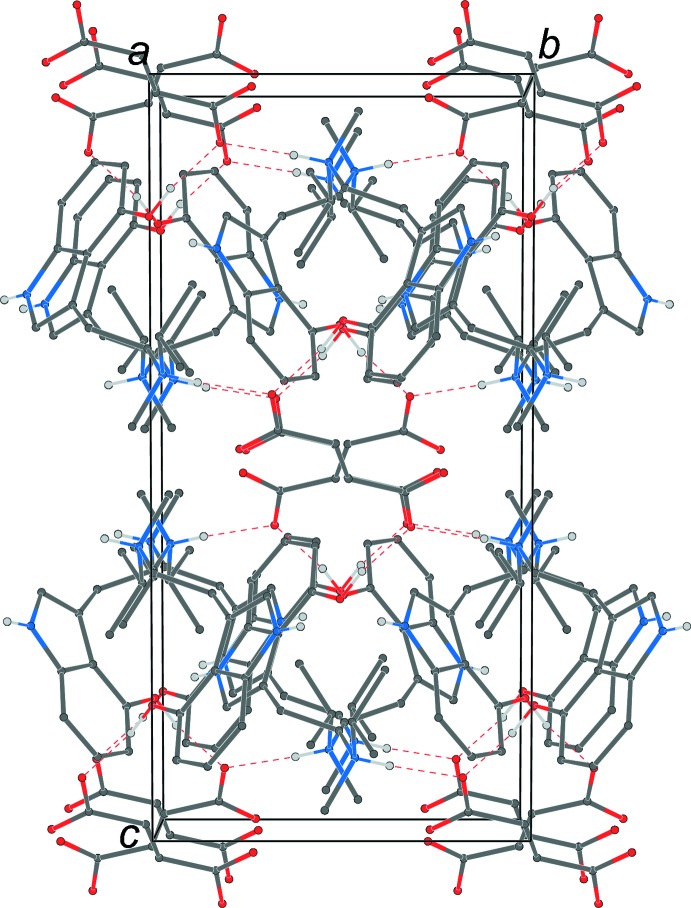
The crystal packing of the title compound, viewed along the *a* axis. The N—H⋯O and O—H⋯O hydrogen bonds (Table 1 ▸) are shown as dashed lines. Hydrogen atoms not involved in hydrogen bonding are omitted for clarity.

There are N2—H2⋯O2 and N2*A*—H2*A*⋯O2 hydrogen bonds between the two configurations of the ammonium cations and one fumarate oxygen. These two different N—H⋯O hydrogen bonds, resulting from the disorder, are also likely to be what produces the fumarate disorder. There is an O1—H1⋯O2 hydrogen bond between the phenol hy­droxy group and one fumarate oxygen atom. Two tryptammonium cations and two fumarate anions are joined together through the N—H⋯O and O—H⋯O hydrogen bonds (Fig. 2 ▸), forming rings with graph-set notation 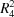
(20) (Etter *et al.*, 1990 ▸). The rings are joined together by two parallel chains along (110). These chains have graph-set notation 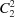
(15) and 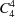
(30). The chains and rings are shown in Fig. 3 ▸. The ions are further linked through N—H⋯π inter­actions between the indole N–H and the aromatic ring of the indole of another tryptammonium ion (Fig. 2 ▸). The hydrogen bonds in the system are outlined in Table 1 ▸. The packing of the compound is shown in Fig. 4 ▸.

## Database survey   

The structure of a number of neutral tryptamines have been reported, including psilocin (Petcher & Weber, 1974 ▸), psilocybin (Weber & Petcher, 1974 ▸), bufotenine (Falkenberg, 1972*b* ▸), DMT (Falkenberg, 1972*a* ▸) and MPT (Chadeayne, Golen & Manke, 2019*b* ▸). A series of one-to-one tryptammonium hydro­fumarate salts have been structurally characterized, including psilacetin (Chadeayne *et al.*, 2019*c* ▸), miprocin and MiPT (Chadeayne, Pham *et al.*, 2019*a* ▸). As discussed above, the two-to-one tryptammonium/fumarate salt of 4-AcO-DMT was previously prepared and its structure reported (Chadeayne, Golen & Manke, 2019*a* ▸). The only other reported two-to-one tryptammonium fumarate salt was that of 4-HO-DPT, or procin (Chadeayne, Pham *et al.*, 2019*b* ▸). The metrical parameters of the tryptammonium cations of 4-HO-MiPT are comparable to those observed for the other reported tryptamine structures.

## Synthesis and crystallization   

61.2 mg of 4-HO-MiPT fumarate were dissolved in 10 mL of deionized water. 29.3 mg of lead(II) acetate was dissolved in 2 mL of deionized water and then added to the tryptamine solution. After sonication, a white precipitate formed. The powder was removed *via* vacuum filtration. The solvent was removed from the resulting solution *in vacuo* to yield a sticky powder. The powder was recrystallized from methanol to yield single crystals suitable for X-ray diffraction.

## Refinement   

Crystal data, data collection and structure refinement details are summarized in Table 2 ▸. Hydrogen atoms H1, H1*A*, H2 and H2*A* were found from a difference- Fourier map and were refined isotropically, using *DFIX* restraints with N—H distances of 0.87 (1) Å and an O—H distance of 0.88 (1) Å. Isotropic displacement parameters were set to 1.2*U*_eq_ of the parent indolic nitro­gen atom and 1.5*U*_eq_ of the parent oxygen atom and the parent ammonium nitro­gen atoms. All other hydrogen atoms were placed in calculated positions with appropriate carbon–hydrogen bond lengths: (*sp^2^*) 0.95 Å, (CH_3_) 0.98 Å, (CH_2_) 0.99 Å and (CH) 1.00 Å. Isotropic displacement parameters were set to 1.2*U*_eq_(C) for *sp^2^*, CH and CH_2_ parent carbon atoms and 1.5*U*_eq_(C-meth­yl). Atoms N2 and C11–C14 were modeled as being disordered over two sets of sites [0.753 (7):0.247 (7)] and refined with *SAD*I (0.03) restraints on C—C(meth­yl) and N—C(meth­yl) bonds to maintain consistent bond lengths in the disorder. Oxygen atom O3 was also modeled as disordered over two sites [0.73 (8):0.27 (8)].

## Supplementary Material

Crystal structure: contains datablock(s) I. DOI: 10.1107/S2056989020002923/zl2774sup1.cif

Structure factors: contains datablock(s) I. DOI: 10.1107/S2056989020002923/zl2774Isup2.hkl

Supporting information file. DOI: 10.1107/S2056989020002923/zl2774Isup3.cml

CCDC reference: 1987588

Additional supporting information:  crystallographic information; 3D view; checkCIF report

## Figures and Tables

**Table 1:** Hydrogen-bond geometry (Å, °) *Cg*2 is the centroid of the C1–C6 ring.

*D*—H⋯*A*	*D*—H	H⋯*A*	*D*⋯*A*	*D*—H⋯*A*
O1—H1⋯O2	0.89 (1)	1.75 (1)	2.618 (2)	165 (2)
N2—H2⋯O2^i^	0.88 (1)	1.85 (1)	2.730 (5)	175 (3)
N2*A*—H2*A*⋯O2^i^	0.87 (1)	1.89 (4)	2.727 (12)	160 (11)
N1—H1*A*⋯*Cg*2^ii^	0.87 (1)	2.78 (2)	3.552 (3)	148 (2)

Symmetry codes: (i) 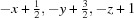; (ii) 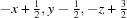.

**Table 2:** Experimental details

Crystal data	
Chemical formula	C_14_H_21_N_2_O^+^·C_2_HO_2_^−^
*M*_r_	290.35
Crystal system, space group	Monoclinic, *C*2/*c*
Temperature (K)	200
*a*, *b*, *c* (Å)	19.770 (13), 9.477 (6), 17.620 (12)
β (°)	105.78 (2)
*V* (Å^3^)	3177 (4)
*Z*	8
Radiation type	Mo *K*α
μ (mm^−1^)	0.08
Crystal size (mm)	0.25 × 0.2 × 0.1

Data collection	
Diffractometer	Bruker D8 Venture CMOS
Absorption correction	Multi-scan (*SADABS*; Bruker, 2018 ▸)
*T*_min_, *T*_max_	0.692, 0.745
No. of measured, independent and observed [*I* > 2σ(*I*)] reflections	39417, 2890, 2007
*R*_int_	0.086
(sin θ/λ)_max_ (Å^−1^)	0.606

Refinement	
*R*[*F* ^2^ > 2σ(*F* ^2^)], *wR*(*F* ^2^), *S*	0.047, 0.105, 1.06
No. of reflections	2890
No. of parameters	265
No. of restraints	11
H-atom treatment	H atoms treated by a mixture of independent and constrained refinement
Δρ_max_, Δρ_min_ (e Å^−3^)	0.15, −0.13

Computer programs: *APEX3* and *SAINT* (Bruker, 2018 ▸), *SHELXT2014* (Sheldrick, 2015*a* ▸), *SHELXL2018* (Sheldrick, 2015*b* ▸), *OLEX2* (Dolomanov *et al.*, 2009 ▸), and *publCIF* (Westrip, 2010 ▸).

## supplementary crystallographic information

### Crystal data

**Table:**

C_14_H_21_N_2_O^+^·C_2_HO_2_^−^	*F*(000) = 1248
*M**_r_* = 290.35	*D*_x_ = 1.214 Mg m^−^^3^
Monoclinic, *C*2/*c*	Mo *K*α radiation, λ = 0.71073 Å
*a* = 19.770 (13) Å	Cell parameters from 5659 reflections
*b* = 9.477 (6) Å	θ = 2.6–23.3°
*c* = 17.620 (12) Å	µ = 0.08 mm^−^^1^
β = 105.78 (2)°	*T* = 200 K
*V* = 3177 (4) Å^3^	Block, colourless
*Z* = 8	0.25 × 0.2 × 0.1 mm

### Data collection

**Table:**

Bruker D8 Venture CMOS diffractometer	2007 reflections with *I* > 2σ(*I*)
φ and ω scans	*R*_int_ = 0.086
Absorption correction: multi-scan (SADABS; Bruker, 2018)	θ_max_ = 25.5°, θ_min_ = 2.6°
*T*_min_ = 0.692, *T*_max_ = 0.745	*h* = −23→23
39417 measured reflections	*k* = −11→11
2890 independent reflections	*l* = −21→21

### Refinement

**Table:**

Refinement on *F*^2^	Secondary atom site location: difference Fourier map
Least-squares matrix: full	Hydrogen site location: mixed
*R*[*F*^2^ > 2σ(*F*^2^)] = 0.047	H atoms treated by a mixture of independent and constrained refinement
*wR*(*F*^2^) = 0.105	*w* = 1/[σ^2^(*F*_o_^2^) + (0.0307*P*)^2^ + 2.6574*P*] where *P* = (*F*_o_^2^ + 2*F*_c_^2^)/3
*S* = 1.06	(Δ/σ)_max_ < 0.001
2890 reflections	Δρ_max_ = 0.15 e Å^−^^3^
265 parameters	Δρ_min_ = −0.13 e Å^−^^3^
11 restraints	Extinction correction: SHELXL2018 (Sheldrick, 2015b), Fc^*^=kFc[1+0.001xFc^2^λ^3^/sin(2θ)]^-1/4^
Primary atom site location: iterative	Extinction coefficient: 0.0036 (5)

### Special details

**Table:**

Geometry. All esds (except the esd in the dihedral angle between two l.s. planes) are estimated using the full covariance matrix. The cell esds are taken into account individually in the estimation of esds in distances, angles and torsion angles; correlations between esds in cell parameters are only used when they are defined by crystal symmetry. An approximate (isotropic) treatment of cell esds is used for estimating esds involving l.s. planes.

### Fractional atomic coordinates and isotropic or equivalent isotropic displacement parameters (Å^2^)

**Table:**

	*x*	*y*	*z*	*U*_iso_*/*U*_eq_	Occ. (<1)
O1	0.31856 (7)	0.49585 (15)	0.58406 (9)	0.0493 (4)	
H1	0.3549 (9)	0.553 (2)	0.5887 (14)	0.074*	
N1	0.21977 (11)	0.1682 (2)	0.71648 (12)	0.0605 (5)	
H1A	0.2128 (12)	0.107 (2)	0.7503 (11)	0.073*	
C1	0.27162 (10)	0.33085 (19)	0.65757 (11)	0.0381 (5)	
C2	0.32760 (11)	0.42144 (19)	0.65257 (12)	0.0400 (5)	
C3	0.38761 (11)	0.4290 (2)	0.71573 (13)	0.0505 (6)	
H3	0.425086	0.489562	0.712691	0.061*	
C4	0.39340 (13)	0.3476 (3)	0.78447 (14)	0.0609 (6)	
H4	0.434890	0.354722	0.826879	0.073*	
C5	0.34054 (14)	0.2583 (3)	0.79158 (14)	0.0620 (7)	
H5	0.344946	0.203862	0.837966	0.074*	
C6	0.27957 (12)	0.2504 (2)	0.72753 (13)	0.0487 (6)	
C7	0.17506 (12)	0.1943 (2)	0.64280 (13)	0.0522 (6)	
H7	0.130641	0.150538	0.622100	0.063*	
C8	0.20396 (10)	0.29280 (19)	0.60364 (12)	0.0400 (5)	
C9	0.17292 (11)	0.33949 (19)	0.51916 (12)	0.0431 (5)	
H9A	0.133346	0.276143	0.494062	0.052*	
H9B	0.209080	0.329163	0.490173	0.052*	
C10	0.14642 (10)	0.49194 (19)	0.51102 (11)	0.0392 (5)	
H10A	0.112079	0.503834	0.542269	0.047*	0.753 (7)
H10B	0.186480	0.555914	0.533343	0.047*	0.753 (7)
H10C	0.098438	0.494556	0.518071	0.047*	0.247 (7)
H10D	0.177245	0.549775	0.553315	0.047*	0.247 (7)
N2	0.1120 (2)	0.5345 (4)	0.4263 (3)	0.0424 (10)	0.753 (7)
H2	0.1020 (16)	0.6251 (14)	0.427 (2)	0.064*	0.753 (7)
C11	0.16090 (17)	0.5281 (3)	0.37193 (17)	0.0460 (11)	0.753 (7)
H11	0.174245	0.427448	0.366898	0.055*	0.753 (7)
C12	0.1208 (6)	0.5835 (11)	0.2891 (5)	0.075 (2)	0.753 (7)
H12A	0.081724	0.519686	0.265521	0.113*	0.753 (7)
H12B	0.152896	0.587641	0.255495	0.113*	0.753 (7)
H12C	0.102418	0.678046	0.294075	0.113*	0.753 (7)
C13	0.2280 (5)	0.6136 (11)	0.4063 (5)	0.0550 (16)	0.753 (7)
H13A	0.254848	0.570569	0.455889	0.082*	0.753 (7)
H13B	0.215425	0.710610	0.416231	0.082*	0.753 (7)
H13C	0.256526	0.614481	0.368717	0.082*	0.753 (7)
C14	0.0444 (4)	0.4557 (8)	0.3919 (4)	0.0602 (16)	0.753 (7)
H14A	0.012269	0.515034	0.352607	0.090*	0.753 (7)
H14B	0.022643	0.432096	0.434091	0.090*	0.753 (7)
H14C	0.054337	0.368833	0.366817	0.090*	0.753 (7)
N2A	0.1450 (7)	0.5560 (14)	0.4311 (9)	0.050 (3)	0.247 (7)
H2A	0.134 (5)	0.643 (4)	0.439 (7)	0.075*	0.247 (7)
C11A	0.1027 (5)	0.4723 (9)	0.3612 (6)	0.056 (4)	0.247 (7)
H11A	0.129490	0.383119	0.360557	0.067*	0.247 (7)
C12A	0.1043 (18)	0.553 (3)	0.2865 (15)	0.072 (9)	0.247 (7)
H12D	0.152027	0.587736	0.291618	0.108*	0.247 (7)
H12E	0.071726	0.632858	0.279034	0.108*	0.247 (7)
H12F	0.090242	0.489724	0.240840	0.108*	0.247 (7)
C13A	0.0317 (13)	0.428 (3)	0.3716 (17)	0.083 (7)	0.247 (7)
H13D	0.038819	0.359379	0.414764	0.124*	0.247 (7)
H13E	0.003480	0.384461	0.322671	0.124*	0.247 (7)
H13F	0.007061	0.510726	0.383946	0.124*	0.247 (7)
C14A	0.2162 (13)	0.601 (3)	0.4231 (16)	0.048 (5)	0.247 (7)
H14D	0.210287	0.677296	0.384573	0.072*	0.247 (7)
H14E	0.238906	0.520050	0.405164	0.072*	0.247 (7)
H14F	0.245452	0.633016	0.474299	0.072*	0.247 (7)
O2	0.41362 (7)	0.68230 (14)	0.57453 (9)	0.0518 (4)	
C15	0.45985 (11)	0.6662 (2)	0.53625 (14)	0.0489 (5)	
C16	0.47710 (10)	0.5170 (2)	0.51944 (13)	0.0438 (5)	
H16	0.453512	0.442783	0.538017	0.053*	
O3	0.4846 (16)	0.7662 (12)	0.5058 (18)	0.079 (4)	0.73 (8)
O3A	0.5040 (17)	0.762 (4)	0.537 (4)	0.066 (7)	0.27 (8)

### Atomic displacement parameters (Å^2^)

**Table:**

	*U*^11^	*U*^22^	*U*^33^	*U*^12^	*U*^13^	*U*^23^
O1	0.0463 (9)	0.0440 (8)	0.0593 (9)	−0.0065 (7)	0.0175 (7)	0.0114 (7)
N1	0.0821 (14)	0.0515 (12)	0.0562 (13)	−0.0066 (11)	0.0332 (11)	0.0146 (10)
C1	0.0498 (12)	0.0287 (10)	0.0412 (11)	0.0039 (9)	0.0215 (10)	−0.0007 (8)
C2	0.0464 (12)	0.0298 (10)	0.0474 (12)	0.0064 (9)	0.0189 (10)	−0.0015 (9)
C3	0.0466 (13)	0.0467 (12)	0.0581 (14)	0.0043 (10)	0.0145 (12)	−0.0041 (11)
C4	0.0633 (15)	0.0632 (15)	0.0520 (14)	0.0116 (13)	0.0085 (12)	−0.0056 (12)
C5	0.0827 (18)	0.0618 (15)	0.0435 (14)	0.0132 (14)	0.0206 (14)	0.0069 (11)
C6	0.0628 (15)	0.0419 (12)	0.0475 (13)	0.0043 (11)	0.0251 (12)	0.0032 (10)
C7	0.0583 (14)	0.0436 (12)	0.0600 (15)	−0.0069 (10)	0.0253 (12)	0.0032 (11)
C8	0.0485 (12)	0.0290 (10)	0.0485 (12)	0.0010 (8)	0.0234 (10)	0.0018 (9)
C9	0.0504 (12)	0.0305 (10)	0.0503 (12)	−0.0006 (9)	0.0168 (10)	−0.0035 (9)
C10	0.0436 (11)	0.0336 (10)	0.0454 (12)	0.0016 (9)	0.0204 (10)	−0.0011 (9)
N2	0.048 (2)	0.0347 (16)	0.0467 (19)	0.0054 (18)	0.016 (2)	0.0011 (13)
C11	0.063 (3)	0.0365 (16)	0.0449 (19)	0.0035 (15)	0.0246 (17)	−0.0003 (13)
C12	0.102 (4)	0.074 (6)	0.049 (3)	−0.009 (4)	0.019 (3)	0.009 (3)
C13	0.051 (4)	0.063 (3)	0.059 (3)	0.001 (3)	0.028 (2)	0.010 (3)
C14	0.056 (4)	0.044 (3)	0.074 (3)	−0.016 (2)	0.007 (3)	0.003 (3)
N2A	0.060 (8)	0.034 (6)	0.056 (6)	0.022 (6)	0.017 (7)	−0.001 (5)
C11A	0.059 (8)	0.034 (5)	0.064 (7)	0.004 (5)	0.000 (5)	−0.007 (5)
C12A	0.13 (2)	0.032 (8)	0.054 (10)	0.029 (12)	0.032 (12)	0.011 (6)
C13A	0.048 (10)	0.061 (11)	0.130 (18)	−0.024 (7)	0.007 (11)	−0.035 (10)
C14A	0.049 (9)	0.039 (7)	0.074 (12)	0.011 (6)	0.046 (7)	0.010 (7)
O2	0.0507 (9)	0.0341 (7)	0.0808 (11)	0.0017 (6)	0.0353 (8)	0.0008 (7)
C15	0.0470 (12)	0.0326 (11)	0.0739 (16)	0.0036 (10)	0.0278 (12)	0.0051 (11)
C16	0.0402 (11)	0.0306 (10)	0.0651 (14)	−0.0004 (9)	0.0221 (10)	0.0069 (9)
O3	0.100 (8)	0.0310 (18)	0.137 (9)	0.008 (3)	0.083 (7)	0.017 (4)
O3A	0.057 (10)	0.031 (6)	0.124 (19)	−0.012 (6)	0.048 (11)	0.001 (9)

### Geometric parameters (Å, º)

**Table:**

O1—H1	0.885 (10)	C11—C13	1.531 (10)
O1—C2	1.367 (2)	C12—H12A	0.9800
N1—H1A	0.870 (10)	C12—H12B	0.9800
N1—C6	1.385 (3)	C12—H12C	0.9800
N1—C7	1.380 (3)	C13—H13A	0.9800
C1—C2	1.422 (3)	C13—H13B	0.9800
C1—C6	1.421 (3)	C13—H13C	0.9800
C1—C8	1.460 (3)	C14—H14A	0.9800
C2—C3	1.390 (3)	C14—H14B	0.9800
C3—H3	0.9500	C14—H14C	0.9800
C3—C4	1.414 (3)	N2A—H2A	0.874 (11)
C4—H4	0.9500	N2A—C11A	1.511 (18)
C4—C5	1.377 (3)	N2A—C14A	1.51 (2)
C5—H5	0.9500	C11A—H11A	1.0000
C5—C6	1.412 (3)	C11A—C12A	1.53 (2)
C7—H7	0.9500	C11A—C13A	1.52 (2)
C7—C8	1.374 (3)	C12A—H12D	0.9800
C8—C9	1.514 (3)	C12A—H12E	0.9800
C9—H9A	0.9900	C12A—H12F	0.9800
C9—H9B	0.9900	C13A—H13D	0.9800
C9—C10	1.530 (3)	C13A—H13E	0.9800
C10—H10A	0.9900	C13A—H13F	0.9800
C10—H10B	0.9900	C14A—H14D	0.9800
C10—H10C	0.9900	C14A—H14E	0.9800
C10—H10D	0.9900	C14A—H14F	0.9800
C10—N2	1.518 (5)	O2—C15	1.284 (2)
C10—N2A	1.527 (15)	C15—C16	1.503 (3)
N2—H2	0.882 (10)	C15—O3	1.251 (10)
N2—C11	1.536 (5)	C15—O3A	1.26 (3)
N2—C14	1.506 (7)	C16—C16^i^	1.315 (4)
C11—H11	1.0000	C16—H16	0.9500
C11—C12	1.550 (9)		
			
C2—O1—H1	109.0 (16)	C13—C11—C12	111.0 (5)
C6—N1—H1A	124.6 (17)	C11—C12—H12A	109.5
C7—N1—H1A	125.8 (17)	C11—C12—H12B	109.5
C7—N1—C6	109.58 (18)	C11—C12—H12C	109.5
C2—C1—C8	134.38 (18)	H12A—C12—H12B	109.5
C6—C1—C2	118.24 (19)	H12A—C12—H12C	109.5
C6—C1—C8	107.32 (18)	H12B—C12—H12C	109.5
O1—C2—C1	116.68 (18)	C11—C13—H13A	109.5
O1—C2—C3	123.93 (19)	C11—C13—H13B	109.5
C3—C2—C1	119.38 (19)	C11—C13—H13C	109.5
C2—C3—H3	119.7	H13A—C13—H13B	109.5
C2—C3—C4	120.7 (2)	H13A—C13—H13C	109.5
C4—C3—H3	119.7	H13B—C13—H13C	109.5
C3—C4—H4	119.1	N2—C14—H14A	109.5
C5—C4—C3	121.8 (2)	N2—C14—H14B	109.5
C5—C4—H4	119.1	N2—C14—H14C	109.5
C4—C5—H5	121.2	H14A—C14—H14B	109.5
C4—C5—C6	117.6 (2)	H14A—C14—H14C	109.5
C6—C5—H5	121.2	H14B—C14—H14C	109.5
N1—C6—C1	106.9 (2)	C10—N2A—H2A	100 (8)
N1—C6—C5	130.8 (2)	C11A—N2A—C10	114.2 (10)
C5—C6—C1	122.3 (2)	C11A—N2A—H2A	122 (8)
N1—C7—H7	124.8	C11A—N2A—C14A	113.2 (16)
C8—C7—N1	110.3 (2)	C14A—N2A—C10	114.4 (13)
C8—C7—H7	124.8	C14A—N2A—H2A	92 (7)
C1—C8—C9	128.51 (17)	N2A—C11A—H11A	106.0
C7—C8—C1	105.86 (18)	N2A—C11A—C12A	107.6 (15)
C7—C8—C9	125.42 (19)	N2A—C11A—C13A	111.8 (12)
C8—C9—H9A	108.8	C12A—C11A—H11A	106.0
C8—C9—H9B	108.8	C13A—C11A—H11A	106.0
C8—C9—C10	113.90 (16)	C13A—C11A—C12A	118.5 (19)
H9A—C9—H9B	107.7	C11A—C12A—H12D	109.5
C10—C9—H9A	108.8	C11A—C12A—H12E	109.5
C10—C9—H9B	108.8	C11A—C12A—H12F	109.5
C9—C10—H10A	108.9	H12D—C12A—H12E	109.5
C9—C10—H10B	108.9	H12D—C12A—H12F	109.5
C9—C10—H10C	109.1	H12E—C12A—H12F	109.5
C9—C10—H10D	109.1	C11A—C13A—H13D	109.5
H10A—C10—H10B	107.8	C11A—C13A—H13E	109.5
H10C—C10—H10D	107.8	C11A—C13A—H13F	109.5
N2—C10—C9	113.2 (2)	H13D—C13A—H13E	109.5
N2—C10—H10A	108.9	H13D—C13A—H13F	109.5
N2—C10—H10B	108.9	H13E—C13A—H13F	109.5
N2A—C10—C9	112.5 (5)	N2A—C14A—H14D	109.5
N2A—C10—H10C	109.1	N2A—C14A—H14E	109.5
N2A—C10—H10D	109.1	N2A—C14A—H14F	109.5
C10—N2—H2	106 (2)	H14D—C14A—H14E	109.5
C10—N2—C11	114.3 (3)	H14D—C14A—H14F	109.5
C11—N2—H2	104 (2)	H14E—C14A—H14F	109.5
C14—N2—C10	112.0 (4)	O2—C15—C16	116.58 (17)
C14—N2—H2	108 (2)	O3—C15—O2	123.5 (5)
C14—N2—C11	111.7 (4)	O3—C15—C16	119.5 (5)
N2—C11—H11	108.6	O3A—C15—O2	120.2 (17)
N2—C11—C12	109.0 (5)	O3A—C15—C16	119.1 (14)
C12—C11—H11	108.6	C15—C16—H16	118.0
C13—C11—N2	110.9 (4)	C16^i^—C16—C15	123.9 (2)
C13—C11—H11	108.6	C16^i^—C16—H16	118.0
			
O1—C2—C3—C4	−179.34 (19)	C8—C1—C2—O1	2.2 (3)
N1—C7—C8—C1	−0.3 (2)	C8—C1—C2—C3	−177.32 (19)
N1—C7—C8—C9	174.91 (18)	C8—C1—C6—N1	−0.1 (2)
C1—C2—C3—C4	0.1 (3)	C8—C1—C6—C5	178.10 (19)
C1—C8—C9—C10	−74.2 (2)	C8—C9—C10—N2	−176.9 (2)
C2—C1—C6—N1	−177.84 (17)	C8—C9—C10—N2A	155.6 (6)
C2—C1—C6—C5	0.4 (3)	C9—C10—N2—C11	−61.5 (3)
C2—C1—C8—C7	177.5 (2)	C9—C10—N2—C14	66.9 (5)
C2—C1—C8—C9	2.4 (3)	C9—C10—N2A—C11A	55.6 (9)
C2—C3—C4—C5	0.1 (3)	C9—C10—N2A—C14A	−77.0 (14)
C3—C4—C5—C6	−0.1 (3)	C10—N2—C11—C12	−176.2 (5)
C4—C5—C6—N1	177.6 (2)	C10—N2—C11—C13	−53.7 (5)
C4—C5—C6—C1	−0.1 (3)	C10—N2A—C11A—C12A	178.0 (14)
C6—N1—C7—C8	0.3 (3)	C10—N2A—C11A—C13A	46.2 (17)
C6—C1—C2—O1	179.16 (17)	C14—N2—C11—C12	55.2 (6)
C6—C1—C2—C3	−0.4 (3)	C14—N2—C11—C13	177.7 (6)
C6—C1—C8—C7	0.3 (2)	C14A—N2A—C11A—C12A	−49 (2)
C6—C1—C8—C9	−174.77 (18)	C14A—N2A—C11A—C13A	179.3 (19)
C7—N1—C6—C1	−0.1 (2)	O2—C15—C16—C16^i^	179.6 (3)
C7—N1—C6—C5	−178.1 (2)	O3—C15—C16—C16^i^	7 (2)
C7—C8—C9—C10	111.7 (2)	O3A—C15—C16—C16^i^	−23 (4)

Symmetry code: (i) −*x*+1, −*y*+1, −*z*+1.

### Hydrogen-bond geometry (Å, º)

*Cg*2 is the centroid of the C1–C6 ring.

**Table:**

*D*—H···*A*	*D*—H	H···*A*	*D*···*A*	*D*—H···*A*
O1—H1···O2	0.89 (1)	1.75 (1)	2.618 (2)	165 (2)
N2—H2···O2^ii^	0.88 (1)	1.85 (1)	2.730 (5)	175 (3)
N2*A*—H2*A*···O2^ii^	0.87 (1)	1.89 (4)	2.727 (12)	160 (11)
N1—H1*A*···*Cg*2^iii^	0.87 (1)	2.78 (2)	3.552 (3)	148 (2)

Symmetry codes: (ii) −*x*+1/2, −*y*+3/2, −*z*+1; (iii) −*x*+1/2, *y*−1/2, −*z*+3/2.
